# Supplementary material for: Morphological and Phylogenetic Analyses Reveal Three New Species of Phyllosticta (Botryosphaeriales, Phyllostictaceae) in China
Source: J Fungi (Basel). 2023 Dec 22;10(1):7. doi: 10.3390/jof10010007 (PMC10816919; doi:10.3390/jof10010007)
Supplement: Supplementary file 1 [file jof-10-00007-s001.zip › Table S1.pdf]

Table S1. Species and GenBank accession numbers of DNA sequences used in this study.

Species	Voucher2	Host/Substrate	Country	GenBank accession number				
				ITS	LSU	tef1	ACT	gapdh
***Phyllosticta capitalensis* species complex**								
P. acaciigena	CPC 28295 [T]	*Acacia suaveolens*	Australia	KY173433	KY173523	NA	KY173570	NA
P. aloeicola	CPC 21020 [T]	*Aloe ferox*	South Africa	KF154280	KF206214	KF289193	KF289311	KF289124
	CPC 21021	*Aloe ferox*	South Africa	KF154281	KF206213	KF289194	KF289312	KF289125
P. ardisiicola	NBRC 102261 [T]	*Ardisia crenata*	Japan	AB454274	NA	NA	AB704216	NA
P.aristolochiicola	BRIP 53316 [T]	*Aristolochia acuminata*	Australia	JX486129	NA	NA	NA	NA
P. azevinhi	MUCC0088	*Ilex pedunculosa*	Japan	AB454302	NA	NA	AB704226	NA
P. beaumarisii	CBS 535.87	*Muehlenbekia adpressa*	Australia	NR_145235	NG_058040	KF766429	KF306232	KF289074
P. brazilianiae	LGMF 330 [T]	*Mangifera indica*	Brazil	JF343572	KF206217	JF343593	JF343656	JF343758
	LGMF 334	*Mangifera indica*	Brazil	JF343566	KF206215	JF343587	JF343650	JF343752
P. capitalensis	CBS 114751	*Vaccinium* sp.	New Zealand	EU167584	EU167584	FJ538407	FJ538465	KF289088
	CBS 128856 [T]	*Stanhopea* sp.	Brazil	JF261465	KF206304	JF261507	JF343647	JF343776
P. carochlae	CGMCC3.17317 [T]	*Caryota ochlandra*	China	KJ847422	NA	KF289178	KF289273	KF289092
P. cavendishii	BRIP 57384	*Musa* cv. *Lady finger*	Australia	KC117644	KU697330	KF009695	KF014059	KU716085
	BRIP 57383	*Musa* cv. *Lady finger*	Australia	KC117643	KU697329	KF009694	KF014058	KU716084
P.cordylinophila	MFLUCC10-0166[T]	*Cordyline fruticosa*	Thailand	KF170287	KF206242	KF289172	KF289295	KF289076
	MFLUCC 12-0014	*Cordyline fruticosa*	Thailand	KF170288	KF206228	KF289171	KF289301	KF289075
P. doitungensis	MFLU 21-0175[T]	*Dasymaschalon obtusipetalum*	Thailand	OK661033	OK661034	OL345581	NA	NA
P. eugeniae	CBS 445.82 [T]	*Eugenia aromatica*	Indonesia	AY042926	KF206288	KF289208	KF289246	KF289139
P. fallopiae	MUCC0113 [T]	*Fallopia japonica*	Japan	AB454307	NA	NA	AB704228	NA
P.guangdongensis	CFCC 58144 [T]	*Viburnum odoratissimum*	China	OQ202160	OQ202170	OQ267758	OQ267764	OQ267770

	CFCC 58766	*Viburnum odoratissimum*	China	OQ202161	OQ202171	OQ267759	OQ267765	OQ267771
	CFCC 58772	*Viburnum odoratissimum*	China	OQ202162	OQ202172	OQ267760	OQ267766	OQ267772
P. ilicis-aquifolii	CGMCC 3.14358 T	*Ilex aquifolium*	China	JN692538	NA	JN692526	JN692514	NA
	CGMCC 3.14359	*Ilex aquifolium*	China	JN692539	NA	JN692527	JN692515	NA
P. maculata	CPC 18347 T	*Musa* cv. *Golygoly pot-pot*	Australia	JQ743570	NA	KF009700	KF014016	NA
	BRIP 46622	*Musa* cv. *Golygoly pot-pot*	Australia	JQ743567	NA	KF009692	KF014013	NA
P. mangiferae	IMI 260576 T	*Mangifera indica*	India	JF261459	KF206222	JF261501	JF343641	JF343748
P. mangifera-indicae	MFLUCC 10-0029T	*Mangifera indica*	Thailand	KF170305	KF206240	KF289190	KF289296	KF289121
P. musaechinensis	GZAAS 6.1247	*Musa* sp.	China	KF955294	NA	KM816639	KM816627	KM816633
	GZAAS 6.1384	*Musa* sp.	China	KF955295	NA	KM816640	KM816628	KM816634
P. musarum	BRIP 57803	*Musa* sp.	Malaysia	JX997138	NA	KF009737	KF014055	NA
	BRIP 58028	*Musa* sp.	Australia	KC988377	NA	KF009738	KF014054	NA
P. oblongifoliae	SAUCC210055	*Garcinia oblongifolia*	China	OM248442	OM232085	OM273890	OM273894	OM273898
	SAUCC210052 T	*Garcinia oblongifolia*	China	OM248445	OM232088	OM273893	OM273897	OM273901
P.paracapitalensis	CPC 26517 T	*Citrus floridana*	Italy	KY855622	KY855796	KY855951	KY855677	KY855735
	CPC 26518	*Citrus floridana*	Italy	KY855623	KY855797	KY855952	KY855678	KY855736
P. parthenocissi	CBS 111645 T	*Parthenocissus quinquefolia*	USA	EU683672	NA	JN692530	JN692518	NA
P.partricuspidatae	NBRC 9466 T	*Parthenocissus tricuspidata*	Japan	KJ847424	NA	KJ847446	KJ847432	KJ847440
	NBRC 9757	*Parthenocissus tricuspidata*	Japan	KJ847425	NA	KJ847447	KJ847433	KJ847441
P. philoprina	CBS 587.69	*Ilex aquifolium*	Spain	KF154278	KF206297	KF289206	KF289250	KF289137
P. phoenicis	CBS 147091	*Phoenix reclinata*	South Africa	MW883442	MW883833	MW890098	MW890031	MW890050
P. pterospermi	SAUCC210104 T	*Pterospermum heterophyllum*	China	OM249954	OM249956	OM273902	OM273904	OM273906

	SAUCC210106	*Pterospermumheterophyllum*	China	OM249955	OM249957	OM273903	OM273905	OM273907
P. rhizophorae	NCYUCC 19-0352^{T}	*Rhizophora stylosa*	China	MT360030	MT360039	NA	MT363248	MT363250
	NCYUCC 19-0358	*Rhizophora stylosa*	China	MT360031	MT360040	NA	MT363249	MT363251
P. saprophytica	SAUCC 1516-2	*the plant saprophytic leaves*	China	OR551459	OR686933	OR621091	OR621084	OR704557
	SAUCC 1516-5	*the plant saprophytic leaves*	China	OR551460	OR686934	OR621093	OR621085	OR704558
P. schimae	CGMCC 3.14354 ^{T}	*Schima superba*	China	JN692534	NA	JN692522	JN692510	JN692506
P. schimicola	CGMCC 3.17319 ^{T}	*Schima superba*	China	KJ847426	NA	KJ847448	KJ847434	KJ854895
	CGMCC 3.17320	*Schima superba*	China	KJ847427	NA	KJ847449	KJ847435	KJ854896
P. styracicola	CGMCC3.14985 ^{T}	*Styrax grandiflorus*	China	JX025040	NA	JX025045	JX025035	JX025030
	CGMCC3.14989	*Styrax grandiflorus*	China	JX025041	NA	JX025046	JX025036	JX025031
P. vitis-rotundifoliae	CGMCC 3.17322 ^{T}	*Vitis rotundifolia*	USA	KJ847428	NA	KJ847450	KJ847436	KJ847442
	CGMCC 3.17321	*Vitis rotundifolia*	USA	KJ847429	NA	KJ847451	KJ847437	KJ847443
Phyllosticta concentrica **species complex**								
P. anhuiensis	CFCC 54840^{T}	*Quercus aliena*	China	OQ202157	OQ202167	OQ267761	OQ267767	OQ267773
	CFCC 55887	*Quercus aliena*	China	OQ202158	OQ202168	OQ267762	OQ267768	OQ267774
	CFCC 58849	*Quercus aliena*	China	OQ202159	OQ202169	OQ267763	OQ267769	OQ267775
P. aspidistricola	NBRC 102244 ^{T}	*Aspidistra elatior*	Japan	AB454314	NA	NA	AB704204	NA
P. aucubae-japonicae	MAFF 236703 ^{T}	*Aucuba japonica*	Japan	KR233300	NA	KR233310	KR233305	NA
P. bifrenariae	CBS 128855 ^{T}	*Bifrenaria harrisoniae*	Brazil	JF343565	KF206209	JF343586	JF343649	JF343744
	CPC 17467	*Bifrenaria harrisoniae*	Brazil	KF170299	KF206260	KF289207	KF289283	KF289138
P.catimbauensis	URM 7672 ^{T}	*Mandevilla catimbauensis*	Brazil	MF466160	MF466163	MF466155	MF466157	NA
	URM 7674	*Mandevilla catimbauensis*	Brazil	MF466161	MF466164	MF466153	MF466158	NA
P. citriasiana	CBS 120486 ^{T}	*Citrus maxima*	Thailand	FJ538360	KF206314	FJ538418	FJ538476	JF343686

P. citriasiana	CBS 120487	*Citrus maxima*	China	FJ538361	KF206313	FJ538419	FJ538477	JF343687
P.citribraziliensis	CBS 100098 T	*Citrus limon*	Brazil	FJ538352	KF206221	FJ538410	FJ538468	JF343691
P. citricarpa	CBS 127454 T	*Citrus limon*	Australia	JF343583	KF206306	JF343604	JF343667	JF343771
P. citrichinensis	ZJUCC 200956 T	*Citrus reticulata*	China	JN791620	NA	JN791459	JN791533	NA
	ZJUCC 2010150	*Citrus maxima*	China	JN791662	NA	JN791514	JN791582	NA
P. citrimaxima	MFLUCC 10-0137 T	*Citrus maxima*	Thailand	KF170304	KF206229	KF289222	KF289300	KF289157
P. concentrica	CBS 937.70	*Hedera helix*	Italy	FJ538350	KF206291	FJ538408	KF289257	JF411745
	CPC 18842 T	*Hedera* sp.	Italy	KF170310	KF206256	KF289228	KF289288	KF289163
P. cussonia	CPC 14873 T	*Cussonia* sp.	South Africa	JF343578	KF206279	JF343599	JF343662	JF343764
	CPC 14875	*Cussonia* sp.	South Africa	JF343579	KF206278	JF343600	JF343663	JF343765
P. elongata	CBS 126.22 T	*Oxycoccus macrocarpos*	USA	FJ538353	NA	FJ538411	FJ538469	KF289164
P. ericarum	CBS 132534 T	*Erica gracilis*	South Africa	KF206170	KF206253	KF289227	KF289291	KF289162
P fujianensis	SAUCC 1366-3 T	*Lonicera japonica*	China	OR551457	OR686935	OR621090	OR621086	OR704555
	SAUCC 1366-5	*Lonicera japonica*	China	OR551458	OR686936	OR621091	OR621087	OR704556
P. gardeniicola	MUCC0117	*Gardenia jasminoides*	Japan	AB454310	NA	NA	AB704230	NA
	MUCC0089	*Gardenia jasminoides*	Japan	AB454303	NA	NA	NA	NA
P. gwangjuensis	CNUFC NJ1-12 T	*Torreya nucifera*	Korea	OK285195	NA	OM038511	OM001471	NA
	CNUFC NJ1-12-1	*Torreya nucifera*	Korea	OK285196	NA	OM038512	OM001472	NA
P. hostae	CGMCC 3.14355 T	*Hosta plantaginea*	China	JN692535	NA	JN692523	JN692511	JN692503
	CGMCC 3.14356	*Hosta plantaginea*	China	JN692536	NA	JN692524	JN692512	JN692504
P.hymenocallidicola	CBS 131309 T	*Hymenocallis littoralis*	Australia	JQ044423	JQ044443	KF289211	KF289242	KF289142
	CPC 19331	*Hymenocallislittoralis*	Australia	KF170303	KF206254	KF289212	KF289290	KF289143
P. hypoglossi	CBS 101.72	*Ruscus aculeatus*	Italy	FJ538365	KF206326	FJ538423	FJ538481	JF343694
	CBS 434.92 T	*Ruscus aculeatus*	Italy	FJ538367	KF206299	FJ538425	FJ538483	JF343695
P. iridigena	CBS 143410 T	*Iris* sp.	South Africa	MG934459	NA	MG934502	MG934466	NA

P. kerriae	MAFF 240047 [T]	*Kerria japonica*	Japan	AB454266	NA	NA	NA	NA
P. kobus	MUCC0049	*Magnolia kobus*	Japan	AB454286	NA	NA	AB704221	NA
P. ophiopogonis	KACC 47754	*Ophiopogon japonicus*	South Korea	KP197057	NA	NA	NA	NA
	LrLF11	*Lycoris radiata*	China	MG543713	NA	NA	NA	NA
P.paracitricarpa	CPC 27169 [T]	*Citrus limon*	Greece	KY855635	KY855809	KY855964	KY855690	KY855748
	ZJUCC 200933	*Citrus sinensis*	China	JN791626	KY855813	JN791468	JN791544	KY855752
P. pilospora	MUCC 2912a [T]	*Chamaecyparis pisifera* var. *plumose*	Japan	LC542597	LC543423	LC543445	LC543465	NA
P. speewahensis	BRIP 58044 [T]	Orchids	Australia	KF017269	NA	KF017268	NA	NA
P. spinarum	CBS 292.90	*Chamaecyparis pisifera*	France	JF343585	KF206301	JF343606	JF343669	JF343773
P. turpiniae	SAUCC2864-3	*Turpinia arguta*	China	OR551461	OR686931	OR621088	OR704559	OR704553
	SAUCC2864-5	*Turpinia arguta*	China	OR551462	OR686932	OR621089	OR704560	OR704554
P. westeae	BRIP 72390c [T]	*Clerodendrum inerme*	Australia	OP599631	NA	OP627090	NA	NA
Phyllosticta cruenta **species complex**								
P. abieticola	CBS 112067	*Abies concolor*	Canada	KF170306	EU754193	NA	KF289238	NA
P. cornicola	CBS 111639	*Cornus florida*	USA	KF170307	NA	NA	KF289234	NA
P. cruenta	CBS 858.71	*Polygonatum odoratum*	Czech Republic	KF170307	NA	MG934501	MG934465	MG934474
P. cruenta	MUCC0206	*Polygonatum odoratum* var.*pluriflorum*	Japan	AB454331	NA	NA	AB704237	NA
P. cryptomeriae	KACC 48643	*Juniperus chinensis* var. *sargentii*	Not given	MK396559	NA	NA	NA	NA
	MUCC0028	*Cryptomeria japonica*	Japan	AB454271	NA	NA	AB704213	NA
P. foliorum	CBS 447.68 [T]	*Taxus baccata*	Netherlands	KF170309	KF206287	KF289201	KF289247	KF289132
P. gaultheriae	CBS 447.70 [T]	*Gaultheria humifusa*	USA	JN692543	KF206298	JN692531	KF289248	JN692508
P. hakeicola	CBS 143492 [T]	*Hakea* sp.	Australia	MH107907	MH107953	MH108025	MH107984	MH107999

P. hamamelidis	MUCC149	*Hamamelis japonica*	Japan	KF170289	NA	NA	KF289309	NA
P. hubeiensis	CGMCC 3.14986 T	*Viburnum odoratissimim*	China	JX025037	NA	JX025042	JX025032	JX025027
	CGMCC 3.14987	*Viburnum odoratissimim*	China	JX025038	NA	JX025043	JX025033	JX025028
P. illicii	24-1-1 T	*Illicium verum*	China	MF198235	MF198240	MF198237	MF198243	NA
	16-16-1	*Illicium verum*	China	MF198234	MF198239	MF198236	MF198242	NA
P. leucothoicola	MUCC553 T	*Leucothoe catesbaei*	Japan	AB454370	AB454370	NA	KF289310	NA
P. ligustricola	MUCC0024 T	*Ligustrum obtusifolium*	Japan	AB454269	NA	NA	AB704212	NA
P. minima	CBS 585.84 T	*Acer rubrum*	USA	KF206176	KF206286	KF289204	KF289249	KF289135
P. neopyrolae	CPC 21879 T	*Pyrola asarifolia*	Japan	AB454318	AB454318	NA	AB704233	NA
P.pachysandricola	MUCC124 T	*Pachysandra terminalis*	Japan	AB454317	AB454317	NA	AB704232	NA
P. paxistimae	CBS 112527 T	*Paxistima mysinites*	USA	KF206172	KF206320	KF289209	KF289239	KF289140
P.podocarpicola	CBS 728.79 T	*Podocarpus maki*	USA	KF206173	KF206295	KF289203	KF289252	KF289134
P. pyrolae	IFO 32652	*Erica carnea*	Not given	AB041242	NA	NA	NA	NA
P. rubella	CBS 111635 T	*Acer rubrum*	USA	KF206171	EU754194	KF289198	KF289233	KF289129
P.sphaeropsoidea	CBS 756.70	*Aesculus hippocastanum*	Germany	AY042934	KF206294	KF289202	KF289253	KF289133
P. telopeae	CBS 777.97 T	*Telopea speciosissima*	Tasmania	KF206205	KF206285	KF289210	KF289255	KF289141
P. yuccae	CBS 112065	*Yucca elephantipes*	USA	KF206175	NA	NA	KF289237	NA
	CBS 117136	*Yucca elephantipes*	New Zealand	JN692541	KF766385	JN692529	JN692517	JN692507
Phyllosticta owaniana **species complex**								
P. austroafricana	CBS 144593 T	leaf spots of unidentified deciduous tree	South Africa	MK442613	MK442549	MK442704	MK442640	NA
P. carissicola	CPC 25665 T	*Carissa macrocarpa*	South Africa	KT950849	KT950863	KT950879	KT950872	KT950876
P. hagahagaensis	CBS 144592 T	*Carissa bispinosa*	South Africa	MK442614	MK442550	MK442705	MK442641	MK442657
P. owaniana	CBS 776.97 T	*Brabejum stellatifolium*	South Africa	FJ538368	KF206293	FJ538426	KF289254	JF343767
	CPC 14901	*Brabejum stellatifolium*	South Africa	JF261462	KF206303	JF261504	KF289243	JF343766

P. podocarpi	CBS 111646	*Podocarpus falcatus*	South Africa	AF312013	KF206323	KC357671	KC357670	KF289169
	CBS 111647	*Podocarpus lanceolata*	South Africa	KF154276	KF206322	KF289232	KF289235	KF289168
P. pseudotsugae	CBS 111649	*Pseudotsuga menziesii*	USA	KF154277	KF206321	KF289231	KF289236	KF289167
***Phyllosticta rhodorae* species complex**								
P. mimusopisicola	CBS 138899 [T]	*Mimusops zeyheri*	South Africa	KP004447	MH878626	NA	NA	NA
P. rhodorae	CBS 901.69	*Rhododendron* sp.	Netherlands	KF206174	KF206292	KF289230	KF289256	KF289166
Phyllosticta vaccinii species complex								
P. vaccinii	ATCC 46255 [T]	*Vaccinium macrocarpon*	China	KC193585	NA	KC193582	KC193580	KC193583
	LC 2795	*Vitis macrocarpon*	USA	KR233323	NA	NA	NA	NA
P. vacciniicola	CPC 18590 [T]	*Vaccinium macrocarpum*	USA	KF170312	KF206257	KF289229	KF289287	KF289165
**Outgroup**								
B. obtusa	CMW 8232 [T]	Conifers	South Africa	AY972105	NA	DQ280419	AY972111	NA
B. stevensii	CBS 112553 [T]	culture from isotype of *Diplodia mutila*	Not given	AY259093	AY928049	AY573219	NA	NA
